# Chlamydia pneumoniae inclusion membrane protein Cpn0147 interacts with host protein CREB3

**DOI:** 10.1371/journal.pone.0185593

**Published:** 2017-09-28

**Authors:** Xia Zhao, Ping Li, Kang An, Xiaohui Jia, Yongting Cheng, Tianjun Jia

**Affiliations:** Laboratory Medicine College, Hebei North University, Zhang Jiakou, Hebei Province, PR China; University of the Pacific, UNITED STATES

## Abstract

Chlamydiae are Gram-negative obligate intracellular bacteria that cause diseases with significant medical and economic impacts. Like other chlamydial species, *Chlamydia pneumoniae* possesses a unique developmental cycle, the infectious elementary body gains access to the susceptible host cell, where it transforms into the replicative reticulate body. The cytoplasmic vacuole where *Chlamydia pneumoniae* replicates is called an inclusion, which is extensively modified by the insertion of chlamydial effectors known as inclusion membrane proteins (Incs). The *C*. *pneumoniae*-specific inclusion membrane protein (Inc) Cpn0147 contains domains that are predicted to be exposed to the host cytoplasm. To map host cell binding partners of Cpn0147, a yeast two-hybrid system was used to screen Cpn0147 against a HeLa cell cDNA library, which led to the finding that Cpn0147 interacted with the host cell protein cyclic adenosine monophosphate (cAMP)-responsive element (CRE)-binding protein (CREB3)_._ The interaction was validated by co-immunoprecipitation of Cpn0147 with CREB3 from HeLa cells ectopically expressing both. Furthermore, Cpn0147 and CREB3 were co-localised in HeLa cells under confocal fluorescence microscopy. The above observations suggest that CREB3 may directly bind to the cytoplasmic domain of Cpn0147 to mediate the interactions of chlamydial inclusions with host cell endoplasmic reticulum.

## Introduction

*Chlamydia pneumoniae* is a respiratory agent of several significant diseases in humans, such as atherosclerosis and asthma. It is an obligate, intracellular human pathogen characterised by a biphasic developmental cycle[1]. A typical *C*. *pneumoniae* infection starts with the entry of an infectious elementary body (EB) in to the host cell. Then, an EB rapidly differentiates into a non-infectious, replicating, and metabolically active reticulate body (RB). This developmental cycle occurs within a membrane bound vesicle termed an inclusion. Chlamydiae complete their biosynthesis and replication within the inclusion. To maintain a successful intravacuolar growth, *Chlamydia* must exchange both materials and signals with host cells possibly via the inclusion membrane. Inclusion membrane proteins (Incs) have been considered to play critical roles in chlamydial interactions with host cells. During the past decade, extensive efforts were made in identifying Incs and the interaction of Incs with host proteins. For example, Cortes et al. investigated Cpn0585 interactions with multiple Rab GTPases. The Cpn0585 interaction with host Rab GTPases may impact the fitness of the *C*. *pneumoniae* inclusion[2]. Flores et al. identified Cpn1027 interactions with Caprin2 and GSK3β both involved in Wnt signalling pathway[3]. Luo et al. identified the hypothetical protein Cpn0147 as a *C*. *pneumoniae* inclusion membrane protein that was co-localized with host cell endoplasmic reticulum (ER)[4]. However, the host interaction partners of Cpn0147 remain unknown.

In the present study, we used the *C*. *pneumoniae* Cpn0147 as a bait to screen HeLa cDNA library in a yeast two-hybrid analysis and found that the host protein cAMP-responsive element (CRE)-binding protein 3 (CREB3) interacted with Cpn0147. Furthermore, both confocal fluorescence microscopy and co-immunoprecipitation (CoIP) assay confirmed the interaction between Cpn0147 and CREB3. We propose that the CREB3-Cpn0147 binding may mediate *C*. *pneumoniae* interactions with the host cell ER.

## Materials and methods

### Cell culture

HeLa cells (ATCC-CCL23) were grown in DMEM medium supplemented with 15% heat-inactivated foetal bovine serum (FBS), 1% L-glutamine and 1% penicillin and streptomycin solution and at 37°C in an atmosphere of 5% CO_2_.

### Yeast two-hybrid analysis

The yeast two-hybrid screen was performed by using a Matchmaker GAL4 two-hybrid system according to the manufacturer’s protocols (Clontech). Yeast strain Y187 was purchased from Clontech, and *Saccharomyces cerevisiae* strain AH109 was saved in our lab. Yeasts were transformed using the Yeast maker Yeast Transformation System 2 (Clontech), and transformants were selected on synthetically defined (SD) plates lacking appropriate nutrients (Clontech). Yeast matings were performed in accordance with the Yeast Two-Hybrid System as described by Clontech. Plasmids were recovered from yeast using Yeast Plasmid Isolation Kit (OMEGA) and transformed into *Escherichia coli* strain XL1-blue. GAL4 DNA-binding domain (DNA-BD) fusions were constructed in pGBKT7 (Clontech). The carboxyl terminus of Cpn0147 was generated through PCR-amplification of Cpn0147 (450 bp) from *C*. *pneumoniae* AR39 genomic DNA (provided by Dr zhong's laboratory, UTHSCSA) using a forward primer with a *Eco*RI site (5’-CGCCGCGAATTCATGGCTGTTCAATCTATAAAAG-3’) and a reverse primer with a *Bam*HI site (5’-TCTGGATCCCTAACTTCCCGCCCCTGAATTGAG-3’). The carboxyl terminus was then ligated into the *Eco*RI and *Bam*HI sites of pGBKT7. GAL4 activation domain (AD) fusions were constructed in pGADT7-Rec (Clontech). HeLa cDNA library was constructed in pGADT7-Rec GAL4 AD as prey. *S*. *cerevisiae* AH109 (Clontech) translated pGBKT7-Cpn0147 was mixed with S. cerevisiae Y187 translated the HeLa cDNA library in the AD vector pGADT7-Rec (Clontech), then they cultured shakily 24 h in 2 × YPDA / Kan (50 μg/mL) liquid medium. The above cell suspension was coated on SD/-Ade/-His/-Leu/-Trp plates. Interacting clones were selected on SD/-Ade/-His/-Leu/-Trp plates. Emerging colonies were checked for activity of the second reporter gene lacZ by performing the colony-lift filter assay using X-Gal (5-bromo-4-chloro-3-indolyl-β-d-galacto-pyranoside) as a substrate. Positive colonies turned blue indicating interaction were picked. The library plasmids were extracted from the selected positive colonies of yeasts and processed for sequencing as described elsewhere. One of the prey plasmids encoded the host protein cAMP-responsive element (CRE)-binding protein 3 (CREB3), which was focused in the current study.

### Mammalian expression constructs and transfections

To achieve optimal Cpn0147 expression in human cells, the full-length DNA sequence of *C*. *pneumoniae* Cpn0147 was optimised for human codon usage (sequence available on demand), synthesised in vitro and then cloned as a *Not*I-*Kpn*I-fragment into pcDNA3.1/myc-His (saved in our lab). The corresponding Cpn0147 (GenBank AE001363.1) DNA fragment was amplified by PCR with a forward primer designed with a 5’ *Not*I site (5’-GGCGGCCGCATGGCTGTTCAATCTATA-3’) and a reverse primer with a 5’ *Kpn*I site (5’-CGGTACCACTTCCCGCCCCTGAATT-3’).

To assemble a mammalian expression vector for CREB3 (GenBank AF468007.1), the corresponding DNA fragment was amplified by PCR with a forward primer designed with a 5’ *Bam*HI site (5’-GCGGATCCACCATGTCTGACAAGAGCGACCTA-3’) and a reverse primer with a 5’ *Eco*RI site (5’-GCGCTCGAGCTAGGCAGCTAACTCAACAGC-3’). Full-length human cDNA served as the template. *Bam*HI-*Eco*RI-digested PCR-fragments were cloned into pcDNA3.1+/flag vector. HeLa cells were transfected with lipo2000 transfection reagent in accordance with the manufacturer’s instructions (Invitrogen) and then incubated for 48 h.

### Co-Immunoprecipitation

HeLa cells were seeded in 10 cm^2^ cell culture dishes until the cells reached approximately 70–80% confluence, and then were transfected with 4 μg of pcDNA3.1/myc-His-Cpn0147 and 4 μg of pcDNA3.1+/flag-CREB3. At 48 h post-transfection, cells were washed three times in cold phosphate buffer solution (PBS), scraped into 1.0 mL of Pierce^®^ IP Lysis Buffer (Thermo Fisher Scientific), agitated for 20 min on ice and then mixed with c-myc agarose. After gentle agitation overnight at 4°C, the agarose were washed three times with 1 mL of PBS, and proteins were eluted into 2× SDS-PAGE sample buffer.

### Immunoblotting

The proteins eluted at 100°C for 5 min were separated through SDS-PAGE on a 12.5% acrylamide gel and then transferred to a nitrocellulose membrane. Membranes were blocked with 5% skim milk in PBST (20 mM Tris-HCl, pH 7.5, 150 mM NaCl, 0.01% (v/v) Tween-20) for 2 h and then incubated overnight at 4°C with rabbit anti-C-myc and mouse anti-flag (a peptide polyclonal antibody produced by Beyotime Biotechnology, China). After washing two times with PBST and once with PBS, the membranes were incubated with horseradish peroxidase-conjugated goat anti-mouse and anti-rabbit secondary antibodies (KeyGEN BioTECH). To detect protein bands, ECL Prime Western Blotting Detection Reagent (KeyGEN BioTECH) was used according to the manufacturer’s instructions.

### Immunofluorescence microscopy

HeLa cells were seeded on cover glasses (NEST Biotechnology) in 6-well plates until the cells reached approximately 70–80% confluence, and were then transfected 2.5 μg of pcDNA3.1/myc-His-Cpn0147 and 2.5 μg of pcDNA3.1+/flag-CREB3, and then incubated for 24 h. The transfected cells were washed three times in cold PBS and then fixed with acetone for 10 min. Cells were washed three times with PBS and then blocked overnight at 4°C with PBS containing 10% FBS. Flag-CREB3 was immunostained with mouse anti-flag, and myc-His-Cpn0147 was immunostained with rabbit anti-c-myc antibody by incubating for 2 h at room temperature. After washing three times with PBS, the cells were incubated with Alexa Fluor^®^488 goat anti-rabbit and cy3 goat anti-mouse secondary antibodies for 2 h under dark conditions. All samples were additionally stained with Hoechst and then extensively washed with PBS. The cells on the coverslips were mounted through fluorescence decay resistant medium, and then turned over onto the glass slide. The fluorescence was observed with a confocal laser scanning microscope (C1-Si, Nikon, Japan).

## Results

### Identification of Cpn0147 interaction with cellular proteins by yeast two-hybrid screening

We employed yeast two-hybrid (YTH) system to search for specific interactions of Cpn0147 with eukaryotic target proteins. The expression of full-length Cpn0147 from *C*. *pneumoniae* fused to the GAL4 DNA-BD in pGBKT7 (Clontech) in *S*. *cerevisiae* AH109 (Clontech) did not activate any of the reporter genes (HIS3 or lacZ). Therefore, this strain was mated with *S*. *cerevisiae* Y187 expressing the normalised HeLa Matchmaker cDNA library in the AD vector pGADT7-Rec (Clontech). HeLa cDNA-expressing plasmids from clones grown on high-stringency medium were isolated and retransformed into competent yeast expressing BD-Cpn0147. Emerging colonies were checked for activity of the reporter gene lacZ, and the positive clones turned blue. For positive clones, HeLa cDNA-expressing plasmids were recovered from these strains, propagated in *E*. *coli* and then sequenced to identify coding sequences. Two isolates were obtained containing inframe-coding sequences for cAMP-responsive element (CRE)-binding protein (CREB3) and acyl-CoA-binding domain-containing protein-3 (ACBD3). Although the potential interaction of Cpn0147 with ACBD3 could also be interesting and deserve further investigation, we only focused on isolating human CREB3 in the current study. CREB3 is a transmembrane protein anchored in the endoplasmic reticulum (ER). CREB3 can modulate ER stress and degrade ER stress-associated protein. To validate the interaction, *S*. *cerevisiae* Y187 expressing pGBKT7-Cpn0147 and *S*. *cerevisiae* AH109 expressing pGADT7-CREB3 were mated and selected on plates with selection medium containing SD/-Ade/-His/-Leu/-Trp, which favoured the growth of positive clones. These observations indicate that *C*. *pneumoniae* Cpn0147 specifically interacted with CREB3 in the YTH system.

### Co-immunoprecipitation of Cpn0147 with CREB3 for validating the interactions between these two

**Fig 1:**
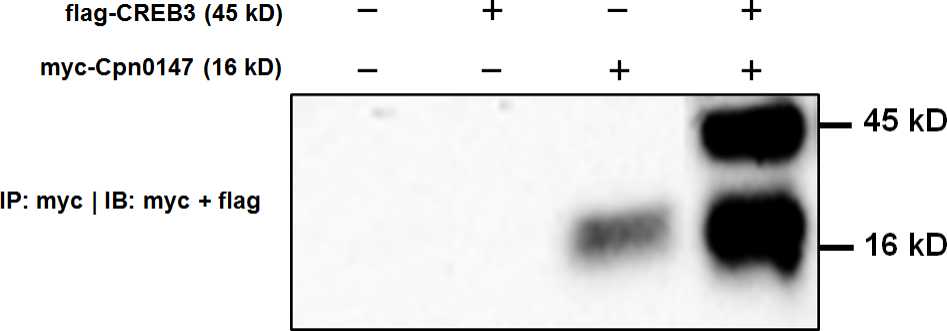
CREB3 interacts with Cpn0147 by immunoprecipitation assay. HeLa cells were transfected with expression constructs encoding myc-tagged Cpn0147 and Flag-tagged CREB3. The cells were lysed 48 h after transfection and subjected to immunoprecipitation with anti-myc antibody. The immunoprecipitation (IP) complexes were analyzed by immunoblotting using both anti-flag and anti-myc antibodies together.

To independently confirm the interaction between Cpn0147 and CREB3, myc-tagged Cpn0147 (containing a 450-bp target gene) and flag-tagged CREB3 (containing a 1113-bp target gene) recombinant plasmids were co-transfected into HeLa cells. After protein expression, cell lysates were made and subjected to immunoprecipitation (IP) with anti-myc antibody. The IP complexes were analyzed by immunoblotting using both anti-Flag and anti-myc antibodies. As shown in Fig 1, the anti-myc antibody both precipitated myc-Cpn0147 (16 kD) and co-precipitated Flag-CREB3 (45 kD), suggesting that there is a direct interaction between CREB3 and Cpn0147.

### Co-localization of Cpn0147 with CREB3 in HeLa cells that ectopically express both proteins

**Fig 2:**
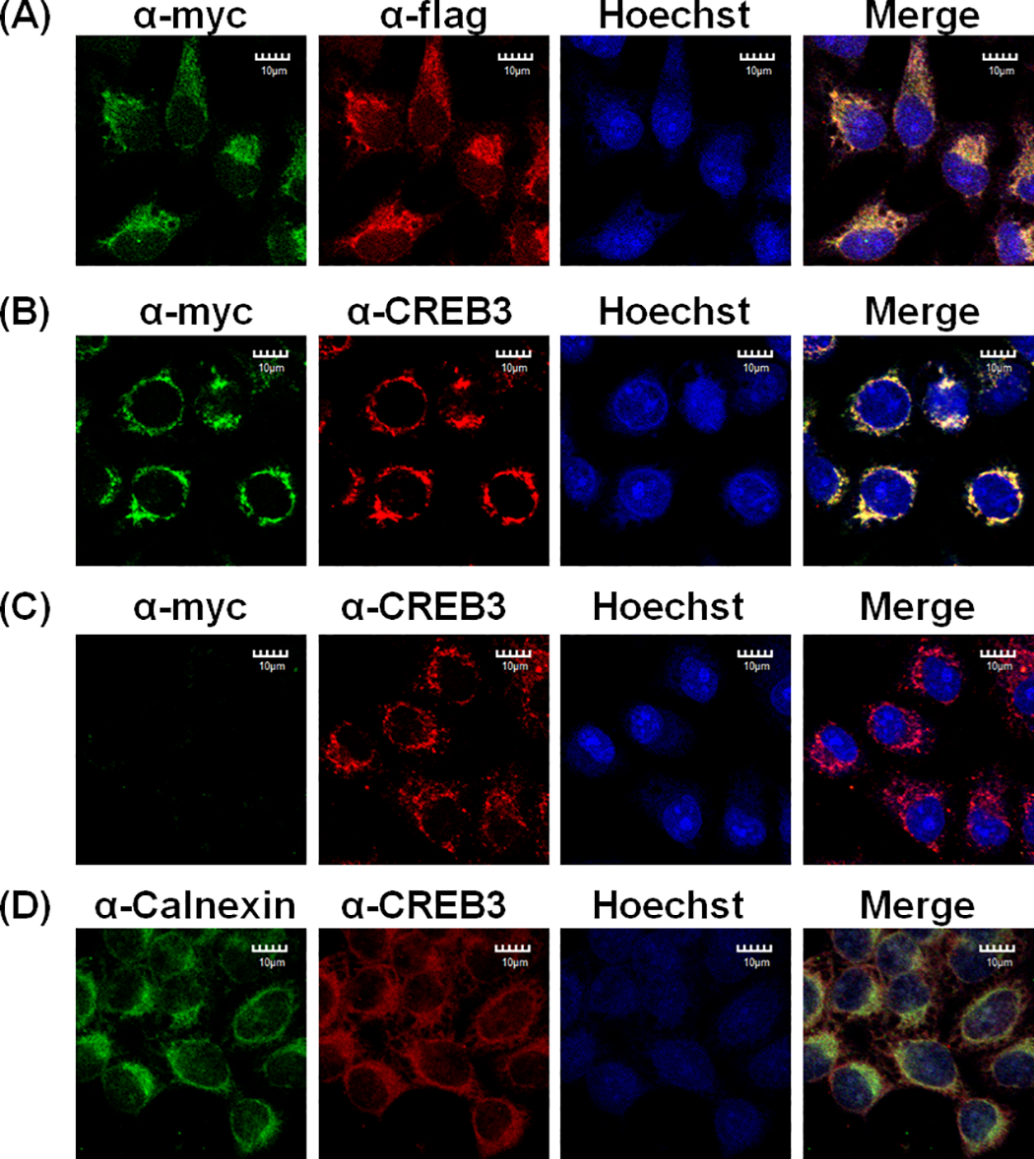
Cpn0147 colocalized with CREB3 by immunofluorescence assay. (A) HeLa cells were transfected with expression plasmids encoding myc-Cpn0147 and flag-CREB3. The cells were then fixed for an immunofluorescence assay to detect Cpn0147 (green) and CREB3 (red) with anti-myc and anti-flag antibodies, respectively, 48 h after transfection. (B) HeLa cells were transfected with expression plasmids encoding myc-tagged Cpn0147. And then Cpn0147 (green) and endogenous CREB3 (red) were detected with anti-myc and anti-CREB3 antibodies, respectively. (C) An empty vector containing c-myc (green) was co-stained with endogenous CREB3 (red) using anti-myc and anti-CREB3 antibodies after transfection. (D) Endogenous calnexin (green) and CREB3 (red) were detected with anti-calnexin and anti-CREB3 antibodies, respectively. Hoechst staining (blue) indicates the locations of the cell nuclei. Fluorescent images were acquired with a confocal laser scanning microscope.

Cell lysis might provide the opportunity for the co-expressed myc-Cpn0147 and flag-CREB3 to interact with each other. To evaluate whether these two proteins interacted with each other inside cells before cell lysis, we examined whether Cpn0147 colocalized with CREB3 in intact cells. After two recombination plasmids, pcDNA3.1/myc-His-Cpn0147 and pcDNA3.1+/flag-CREB3, were co-transfected into HeLa cell, the transfected cells were processed for immunofluorescence labelling and observed by confocal microscopy. As shown in Fig 2A, Cpn0147 colocalized mostly with CREB3 in the cytosol of HeLa cells. Furthermore, we also investigated the spatial relationship between transfected myc-Cpn0147 and cellular endogenous CREB3 in host cells, and the similar results were observed (Fig 2B). In addition, no colocalization was observed with the empty vector alone (Fig 2C). Calnexin is a membrane-bound lectin and a molecular chaperone that located in the endoplasmic reticulum (ER)[5]. Colocalization of CREB3 with calnexin further verified that CREB3 anchoring in the ER (Fig 2D). Collectively, these data suggest that CREB3 may interact with Cpn0147 in live cells, which may mediate *C*. *pneumoniae* interactions with host cell endoplasmic reticulum in favour of *C*. *pneumoniae* intracellular survival.

## Discussion

Chlamydiae complete a productive growth cycle in host cells by utilising a number of host cellular processes, including interactions between host cell molecules and chlamydial products, and most of which are in cytosol of cells. Here, we identified a novel interaction in the endoplasmic reticulum between the *C*. *pneumoniae* inclusion membrane protein Cpn0147 and the host cAMP-responsive element (CRE)-binding protein (CREB3).

*C*. *pneumoniae* are obligate intracellular bacterial pathogens that utilise a developmental cycle to alternate between infectious, metabolically quiescent EBs and non-infectious, metabolically active RBs during a productive growth cycle. Following endocytosis, EBs enter the cell and then multiply in an inclusion, which is wrapped by the host cell membrane. The inclusion acts as a micro-environment for the inclusion of Chlamydia survival through absorbing nutrients and excreting metabolic products. The bacterium communicates with the host cell in the inclusion. The inclusion membrane can avoid inclusion in the lysosomal pathway and other innate immune defences and can permeate to ions and small molecules [6, 7]. The inclusion is segregated from the host endocytic pathway, but most of the underlying effector mechanisms are unknown. Transiently interacting with the Golgi and ER, the inclusion acquires cholesterol and sphingolipids from the secretory pathway to selectively intercept vesicles. Since ER markers also enter the inclusion and associate with luminal bacteria, ER membranes may be additionally metabolised and potentially incorporated into bacterial envelopes to support growth [8]. ER-related BiP/GRP78 plays a key role to restore cells damaged from stressful conditions during the early phase of IFN-γ-induced persistent infection [9]. Chlamydia synthesizes c-di-AMP. STING in the ER, an organelle that innervates the entire cytoplasm, has a greater likelihood of sensing Chlamydial c-di-AMP and translocating to signalling platforms together with TBK1 and IRF3[10]. *Chlamydia trachomatis* effector protein IncD specifically interacts with the non-vesicular ceramide transfer protein (CERT) at ER-Golgi membrane contact sites (MCSs) between *C*. *trachomatis* inclusion membrane and ER tubules, harbouring the VAPA/B proteins [11, 12]. The interaction among IncD-CERT-VAPA/B may provide a specialized metabolic and/or signalling microenvironment for Chlamydial development. Since Cpn0147 was identified as an inclusion membrane protein of *C*. *pneumoniae*, however, the host interaction partners of Cpn0147 were still unclear over the last decade.

CREB3 (also called Luman) was anchored in the endoplasmic reticulum (ER) as a transmembrane protein, modulates ER stress and ER stress-associated protein degradation [13]. Some studies revealed that host protein CREB3 was involved in and also associated with several pathogens. Mirabelli *et al*. found that CREB3/Herp (homocysteine-induced ER protein) pathway limited the increase in cytosolic Ca^2+^ concentration and apoptosis in the early of poliovirus infection [14]. CREB3 bound to human immunodeficiency virus (HIV) protein Tat in order to inhibit assembly of viral particles through blocking the Tat activity [15]. CREB3 was also involved in resistant to lytic infection of herpes simplex virus (HSV) by interacting with human factor C1 (HCF) in the ER, in order to interfere with viral protein VP-16-activated transcription of viral genes [16]. Especially, Reiling *et al*. found that CREB3 was required for the induction of ADP-ribosylation factor 4 (ARF4) involving cellular Golgi stress response, and CREB3-ARF4 signalling cascade was investigated to support the survival of *Chlamydia trachomatis* and *Shigella flexneri* [17]. In our study, we demonstrated the interaction of Cpn0147 and CREB3 for the first time, and it was perfectly possible that this interaction influenced the development of *C*. *pneumoniae* or the host cellular signalling pathway. Unfortunately, we are not able to perform the experiments of infecting cells under the current technology in our lab. The biological significances of CREB3-Cpn0147 interaction in *C*. *pneumoniae*-infected cells need to be further investigated.

In conclusion, our data provided an important insight into searching the host proteins interacting with *C*. *pneumoniae* inclusion membrane protein Cpn0147. Then our finding identified the interaction between Cpn0147 and the host protein CREB3, and further study will be required to elucidate the roles of this interaction.
